# Mitochondrial DNA Copy Number Drives the Penetrance of Acute Intermittent Porphyria

**DOI:** 10.3390/life13091923

**Published:** 2023-09-15

**Authors:** Elena Di Pierro, Miriana Perrone, Milena Franco, Francesca Granata, Lorena Duca, Debora Lattuada, Giacomo De Luca, Giovanna Graziadei

**Affiliations:** 1 Foundation IRCCS Ca’ Granda Ospedale Maggiore Policlinico, 20122 Milan, Italy; labporfirie@policlinico.mi.it (M.P.); francesca.granata@policlinico.mi.it (F.G.); lorena.duca@policlinico.mi.it (L.D.); debora.lattuada@policlinico.mi.it (D.L.); giovanna.graziadei@policlinico.mi.it (G.G.); 2 Department of Molecular Medicine, University of Pavia, 27100 Pavia, Italy; milena.franco01@universitadipavia.it; 3 School of Internal Medicine, University of Milan, 20122 Milan, Italy; giacomodeluca29@gmail.com

**Keywords:** acute intermittent porphyria, heme, mtDNA copy number, mitochondrial biogenesis, PERM1, incomplete penetrance

## Abstract

No published study has investigated the mitochondrial count in patients suffering from acute intermittent porphyria (AIP). In order to determine whether mitochondrial content can influence the pathogenesis of porphyria, we measured the mitochondrial DNA (mtDNA) copy number in the peripheral blood cells of 34 patients and 37 healthy individuals. We found that all AIP patients had a low number of mitochondria, likely as a result of a protective mechanism against an inherited heme synthesis deficiency. Furthermore, we identified a close correlation between disease penetrance and decreases in the mitochondrial content and serum levels of PERM1, a marker of mitochondrial biogenesis. In a healthy individual, mitochondrial count is usually modulated to fit its ability to respond to various environmental stressors and bioenergetic demands. In AIP patients, coincidentally, the phenotype only manifests in response to endogenous and exogenous triggers factors. Therefore, these new findings suggest that a deficiency in mitochondrial proliferation could affect the individual responsiveness to stimuli, providing a new explanation for the variability in the clinical manifestations of porphyria. However, the metabolic and/or genetic factors responsible for this impairment remain to be identified. In conclusion, both mtDNA copy number per cell and mitochondrial biogenesis seem to play a role in either inhibiting or promoting disease expression. They could serve as two novel biomarkers for porphyria.

## 1. Introduction

Porphyria refers to a group of rare inherited metabolic disorders caused by the dysfunction of heme synthesis [1,2]. This process takes place in all cells, originating from glycine and succinyl-CoA and progressing through eight enzymatic reactions, with four occurring in the cytoplasm and the remaining four within the mitochondria. The pathway is rigorously regulated at its inception, controlled by the first and rate-limiting enzyme in the pathway, delta-aminolevulinic acid synthase (ALAS; EC 2.3.1.37) [3]. About 75%–80% of the daily-synthesized heme is used for hemoglobin production in the bone marrow, 15% contributes to cytochrome P-450 synthesis in the liver, while the remainder serves vital functions in all cells. Heme acts as an essential cofactor for numerous hemoproteins, among which the most important are mitochondrial cytochromes engaged in redox and respiratory reactions in cells, mainly in the liver and muscles [4].

In erythroblasts, regulatory mechanisms facilitate the production of the substantial heme quantities required for hemoglobin synthesis. Erythroid heme biosynthesis depends on iron availability, which regulates the mRNA translation of erythroid-specific ALAS isoform 2 and serves as a substrate in the pathway’s final stage. Conversely, in all other cells, particularly hepatocytes, heme biosynthesis is influenced by negative feedback control related to the pool of “free” heme. Elevated heme levels within cells suppress the mRNA synthesis of ubiquitous ALAS isoform 1 and impede the enzyme’s transport from the cytosol to the mitochondria [5]. Several factors, including drugs and conditions like infection, stress, and hormones, reduce intracellular heme concentration by stimulating P450 hemoproteins or heme catabolism, relieving ALAS1 suppression. Additionally, fasting, through the activation of peroxisome-proliferator-activated receptor gamma coactivator 1 alpha (PGC-1α), a transcriptional regulator, results in an upsurge in ALAS1 transcription [6,7].

Genetic anomalies associated with heme synthesis enzymes and/or porphyrinogen agents lead to enzymatic deficiencies and blockage at specific enzymatic steps within the pathway. Depending on the affected enzyme, distinct tissue accumulation patterns and subsequent excretion of the porphyrin precursors, delta-aminolevulinic acid (ALA), and porphobilinogen (PBG), and/or porphyrins can occur. These patterns are unique to each porphyria type and correspond to specific clinical manifestations. Existing evidence suggests that the porphyrin precursors cause neuronal damage, while the porphyrins induce skin-related symptoms. Porphyria are classified as hepatic or erythropoietic based on the primary site of expression of the underlying enzyme deficiency. Furthermore, porphyria is categorized as acute when patients experience neurovisceral acute attacks or cutaneous when they exhibit blistering or photosensitivity of the skin [8,9].

Acute intermittent porphyria (AIP; OMIM#176000) is the most common among the acute hepatic forms and arises from haploinsufficiency in the hydroxymethylbilane synthase (*HMBS*) gene, the third enzyme in heme synthesis [10]. In AIP patients, 527 different *HMBS* pathogenic mutations have been reported worldwide (Human Gene Mutation Database, 2021), with an estimated prevalence of approximately 1 carrier per 2000 individuals, among Caucasians [11,12]. However, a notably low penetrance has been observed in this autosomal-dominant inherited disease, with only about 1% of heterozygotes carrying pathogenic mutations experiencing symptoms during their lifetime [13,14]. This underscores the significant role of environmental factors and possibly genetic modifiers.

Acute intermittent porphyria (AIP) is characterized by acute attacks presenting as severe abdominal pain, often accompanied by nausea, vomiting, autonomic dysfunction (tachycardia, elevated systolic blood pressure), and mild cognitive symptoms [15]. Other types of pain, such as back and limb pain, have also been reported during attacks [16]. Untreated or worsened by additional provoking factors, acute attacks can progress to acute neuropathy and/or life-threatening encephalopathy [17,18]. The neurological deficits, including progressive muscle weakness, typically manifest after 3–21 days of abdominal pain and dysautonomia [19,20]. Symptoms arise due to the hepatic production of neurotoxic porphyrin precursors, ALA and PBG, which are also detected in large quantities in urine [21]. The reduction in the enzymatic activity of the encoded porphobilinogen deaminase (PBGD, EC 2.5.1.61; MIM#609806), combined with other triggering factors (nutritional, hormonal, drugs, or infections), leads to a critical deficiency in the “heme regulatory pool” or stimulates heme synthesis through PGC-1α activation, which results in ALA and PBG accumulation and subsequent attacks [22,23].

While most AIP patients experience sporadic attacks throughout their lives, about 2–5% suffer from recurrent attacks, necessitating frequent hospitalizations. Among recurrent patients, 90% are women, typically experiencing onset after puberty [15,24]. Additionally, some AIP patients (high excreters) become clinically asymptomatic after a symptomatic period despite permanently elevated heme precursor concentrations [25]. Recent studies have also reported an increased prevalence of chronic symptoms in latency phases, marked by chronic neuropathic pain between the attacks and characterized by small fiber neuropathy and peripheral and/or central sensitization [16,26]. An oligogenic inheritance model with environmental modifiers has been proposed to better explain the low penetrance and high variability in the clinical presentation of this autosomal dominant disease [27]. However, conclusive evidence regarding the involvement of genetic and epigenetic factors remains elusive.

Hepatic transcriptomic analysis in AIP animal models has revealed the dysregulation of genes associated with mitochondrial biogenesis [28]. Mitochondrial biogenesis involves the growth and division of pre-existing mitochondria and serves as an adaptive mechanism in response to various external stimuli and changes in physiological states requiring increases in rates of ATP utilization [29]. Changes in mitochondrial mass have been documented in both normal and disease states. Thyroid hormones have long been associated with increased mitochondrial mass and elevated expression of key metabolic enzymes and respiratory cytochromes in responsive tissues. Mitochondrial proliferation also occurs in the brown fat during adaptive thermogenesis [30]. It is well established that mitochondrial biogenesis increases in muscle cells following exercise, intensive physical activity, or in response to chronic contractile activity induced by continuously working muscles, such as in the heart [29]. Moreover, proliferation of abnormal mitochondria has been observed in the muscle fibers of patients with specific mitochondrial myopathies, with similar changes demonstrated in mouse models of mitochondrial disease [31]. These observations suggest that the individual responsiveness to endogenous or environmental stressors and/or potential disruptions in the molecular machinery governing mitochondrial DNA replication and mitochondrial biogenesis may play a modifying role contributing to incomplete penetrance in porphyria.

Moreover, functional anomalies in mitochondrial respiratory chain complexes and oxidative phosphorylation have been reported in AIP animal models [32,33] and porphyria patients [34], underscoring the potential involvement of energy supply impairment in the clinical presentation of the disease. However, changes in the number of mitochondria have not been evaluated.

In this study, we conducted a relative quantification of the mitochondrial DNA (mtDNA) copy number in the peripheral white blood cells to indirectly estimate the average mitochondrial content per cell in symptomatic (S) versus asymptomatic (AS) AIP patients, comparing these results with those of healthy individuals (controls (CTRL)).

## 2. Materials and Methods

### 2.1. Subjects

A total of 34 patients diagnosed with AIP and 37 healthy individuals, matched for age and sex, were included in the analysis. All participants provided written informed consent in accordance with the Declaration of Helsinki guidelines. Diagnosis of the patients was based on the presence of a pathogenic gene variant in the *HMBS* gene, and their classification followed the new definitions proposed by experts [35]. The “symptomatic category” included both sporadic and recurrent active patients, along with symptomatic chronic high excreters. The “asymptomatic category” included both latent at-risk and in-remission patients, plus asymptomatic high excreters (Table 1).

### 2.2. Quantification of mtDNA Copy Number

Total DNA was isolated from the peripheral white blood cells using a Blood DNA Purification System kit in a Maxwell 16 automatic instrument (Promega Corporation, Madison, WI, USA). Assessment of DNA sample quantity and integrity was performed employing a NanoDrop 1000 photometer and a dsDNA broad-range assay kit for the Qubit fluorimeter (ThermoFisher, Waltham, MA, USA), respectively. Real-time relative quantification was executed utilizing an ABI prism 7500 system (Applied Biosystems, Foster City, CA, USA) under standard cycling conditions. Each sample, comprising 150 ng of total DNA, underwent amplification for both the *RPPH1* nuclear gene (utilized as a genomic copy number reference) and the *7S* mitochondrial DNA (located in the mitochondrial replication start site within the D-loop non-coding region [36]), with separate wells to ensure optimal amplification efficiency. PCR was conducted in duplicate with a total volume of 20 µL. The reagents employed included 1× TaqMan fast advance PCR master mix (Cat. No. 4444963), along with 1 × VIC-labeled *RNase P* assay probe (Cat. No. 4403326) or 1 × FAM-labeled *MT-7S* TaqMan probe (Hs01596861__s1), respectively. Data analysis was conducted through the comparative Ct method, where Ct indicates the cycle number at which fluorescence initially exceeds the threshold. The Δ cycle threshold (ΔCt) values were calculated for each sample by subtracting the D-loop target gene values from the RNase P reference gene values, thus facilitating the normalization of mtDNA in relation to nuclear DNA. Subsequently, for each sample, the 2×2^−ΔCT^ was computed, with 2 representing the haploid-to-diploid conversion factor [37]. Each sample underwent analysis in no fewer than three independent experiments, with the results being graphically presented as the average of the values obtained in each experimental run.

### 2.3. Evaluation of the Integrity of mtDNA

We conducted qPCR experiments utilizing specific TaqMan assays for the mitochondrial genes *ND4* (Hs02596876_g1), *CYTB* (Hs02596867_s1), and *ND1* (Hs02596873_s1). *ND4* is susceptible to common deletions in cases of mitochondrial damage [38], while *CYTB* and *ND1* serve as indicators for the initiation and termination of mitochondrial replication, respectively [39]. Relative abundance comparisons were made for each mitochondrial gene with its nuclear counterpart, and, subsequently, the ratios of *ND4/CYTB* and *ND4/ND1* copy numbers were computed.

### 2.4. Measurement of Heme Levels

Total heme concentrations within patient sera were determined colorimetrically using a QuantiChromTm Heme assay kit DIHM-250 (BioAssay Systems, Hayward, CA, USA), following the manufacturer’s instructions. This direct assay was based on an improved aqueous alkaline solution method wherein the heme-containing solution becomes uniformly colored. The measurement accurately represents total serum heme, including both cell-free heme and heme bound to scavengers and proteins.

### 2.5. Mitochondrial Biogenesis Evaluation by PERM1 Measurement

We measured serum PERM1 (peroxisome proliferator-activated receptor gamma coactivator 1 (PPARGC or PGC-1) and estrogen-related receptor (ERR or ESRR)-induced regulator in muscle 1) using a human uncharacterized protein C1orf170 (chromosome 1 open reading frame 170) ELISA Kit (MBS9322691 by MyBiosource, San Diego, CA, USA). This quantitative sandwich kit is based on the C1orf170 antibody–C1orf170 antigen interactions and an HRP colorimetric detection system to quantify native C1orf170 (PERM1) antigen targets in samples. We followed the manufacturer’s instructions and used MyAssay’s Data Analysis online tool (https://www.myassays.com/, accessed on 1 May 2023) to plot the standard fitting curve and calculate the level of the analyte.

### 2.6. Statistical Analysis

All statistical analyses were carried out using Prism software (version 9.5, GraphPad Software, Boston, MA, USA). For each parameter, descriptive statistics (mean, SD, median, IQR, min, and max) were computed. The data underwent testing for normality and log-normality using the D’Agostino–Pearson, Anderson–Darling, Shapiro–Wilk, and Kolmogorov–Smirnov tests. Between-group comparisons of normally distributed data were conducted using two-tailed unpaired t-tests, with Welch’s correction applied in cases of unequal standard deviation (SD). For non-normally distributed data, between-group comparisons were performed using the Mann–Whitney test. Multiple comparisons were analyzed using either the one-way ANOVA or Kruskal–Wallis test. Statistical significance was defined as *p* < 0.05 for all group differences.

## 3. Results

### 3.1. The mtDNA Copy Number Was Lower in AIP Patients

**Figure 1:**
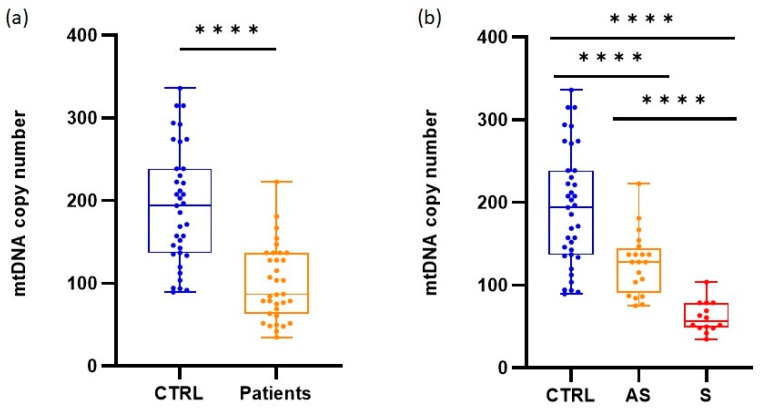
Quantification of mtDNA copy number by qPCR. The figures display the distribution of values obtained with median, interquartile range, minimum, and maximum values. (**a**) Comparison between healthy individuals (CTRL) and total AIP cases (Patients). (**b**) Comparison among healthy individuals, asymptomatic (AS) patients, and symptomatic (S) patients; **** adjusted *p* < 0.0001.

We assessed the average copy number of mtDNA per cell in 37 healthy individuals and 34 AIP patients. The patients exhibited a significantly lower mtDNA content (Welch-corrected *t* = 6.687, *p* < 0.0001) in peripheral white blood cells compared with healthy controls (Figure 1a). The median value (with interquartile range (IQR)) of mtDNA copy number was 194 (136.4–238.9) for healthy individuals and 86.87 (62.91–137.2) for patients. This reduction was more pronounced when the patients were classified into the asymptomatic and symptomatic groups, with median values of 128 (91.32–144.6) and 56.37 (48.44–78.79), respectively (Figure 1b).

**Figure 2:**
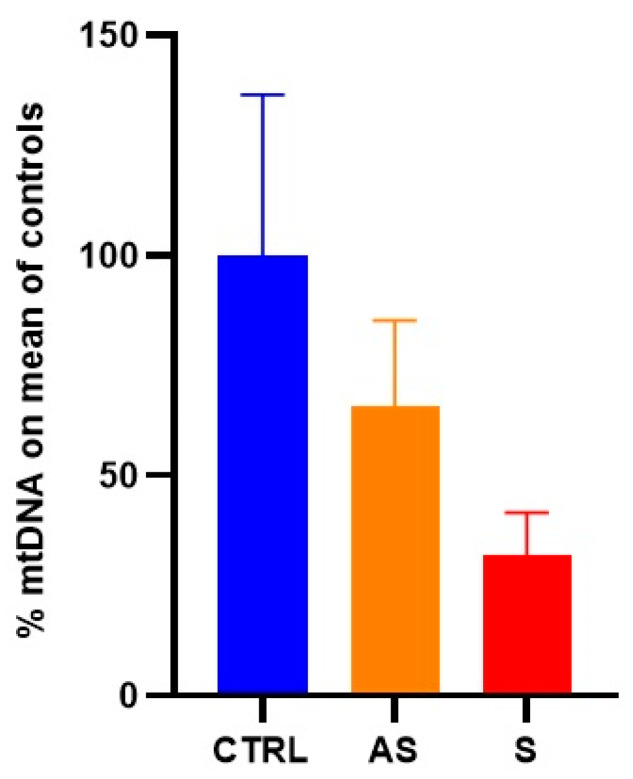
Relative percentage of the mtDNA content. We calculated the ratio of mtDNA copy number to the mean of controls for all individuals analyzed to determine the mean percentage. The panel presents the mean ± SD of mtDNA percentage values obtained for healthy individuals (CTRL), asymptomatic (AS) patients, and symptomatic (S) patients. The statistical significance for reduction is not reported since the same values were used in Figure 1, and the significance remained the same (*p* < 0.0001).

Symptomatic patients showed a 70% reduction in average mtDNA content relative to healthy individuals, while asymptomatic patients exhibited a 35% reduction. The mean values ± SD of the percentage of mtDNA for symptomatic patients, asymptomatic patients, and healthy individuals were 31.88 ± 9.6%, 65.85 ± 19.38%, and 100 ± 36.45%, respectively (Figure 2).

### 3.2. The Integrity of mtDNA Was Not Compromised

**Figure 3:**
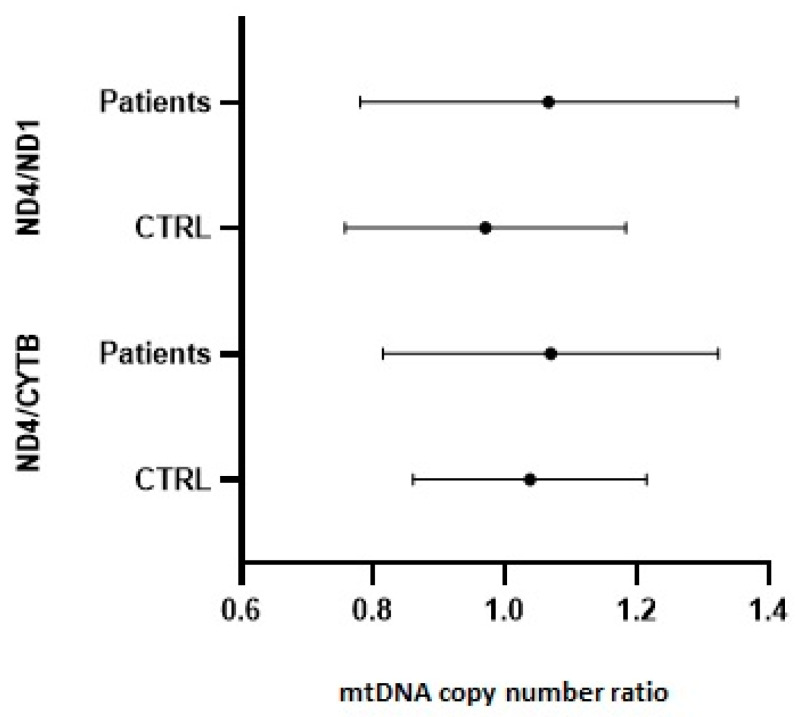
Integrity of mtDNA. The ratio of intact copies of mtDNA (ND4 gene) to the total of mtDNA detected (ND1 or CYTB genes) was calculated for all individuals. The results are presented as the mean ± SD. A value close to 1 (0.8–1.2) indicates that no mtDNA damage occurred. There were no significant differences between healthy individuals (CTRL) and patients.

We assessed the ratio of intact mtDNA (ND4) to total mtDNA (CYTB or ND1) copy number for each sample to exclude possible damage due to mutations. The integrity of the mtDNA was similar in AIP patients and healthy controls, with a ratio ranging from 0.79 to 1.35 and 0.75 to 1.22, respectively. The mean values ± standard deviation (SD) for the ND1 and CYTB genes were 1.066 ± 0.28 vs. 0.96 ± 0.21 and 1.069 ± 0.25 vs. 1.038 ± 0.18, respectively (Figure 3).

### 3.3. Systemic Heme Levels Decreased in AIP Patients

**Figure 4:**
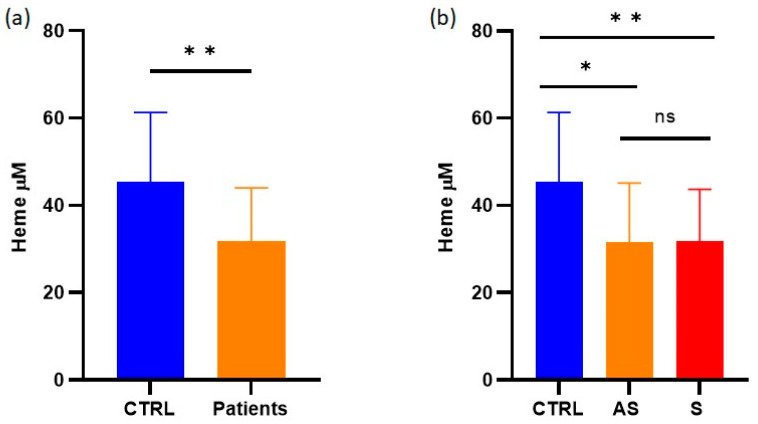
Quantification of heme. The results are graphically presented as the mean ± SD. (**a**) Comparison was performed between healthy individuals (CTRL) and all AIP cases (patients). (**b**) Comparisons were performed among healthy individuals, asymptomatic (AS) patients, and symptomatic (S) patients; ns = not significant, * exact *p* < 0.05, and ** exact *p* < 0.01.

The serum level of cell-free heme, including unbound heme and heme bound to scavengers and proteins, was significantly lower in the AIP cohort than in healthy individuals (31.71 ± 12.31 µM vs. 45.34 ± 15.97 µM, Mann–Whitney U = 113, *p* < 0.01). However, there were no significant differences between asymptomatic and symptomatic patients (Figure 4a,b).

### 3.4. Mitochondrial Biogenesis Was Impaired in Asymptomatic and Symptomatic Patients

**Figure 5:**
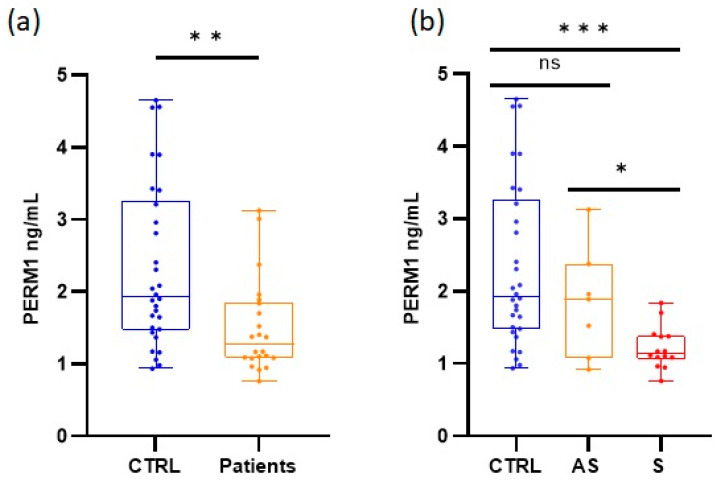
Quantification of PERM1. The figure shows the distribution of values obtained with median, interquartile range, minimum, and maximum values (**a**) Comparison was performed between healthy individuals (CTRL) and total AIP cases (patients). (**b**) Comparisons were made among healthy individuals, asymptomatic (AS) patients, and symptomatic (S) patients; ns = not significant, * *p* < 0.05, ** *p* < 0.01, and *** *p* < 0.001.

The PERM1 factor was analyzed in the serum of a subgroup of 22 AIP patients (8 AS and 14 S) because it is required for PGC1α-induced mitochondrial biogenesis and maximal oxidative capacity in the muscle. Similar to the results of the mtDNA copy number, the AIP cohort exhibited significantly lower levels of PERM1 (*t* = 3.279, *p* < 0.01) than healthy individuals, with median values (and interquartile range) of 1.932 ng/mL (1.470–3.260) and 1.273 ng/mL (1.085–1.851), respectively. This reduction was more pronounced (*p* < 0.001) when the patients were classified into the asymptomatic and symptomatic groups, with values of 1.888 ng/mL (1.077–2.379) and 1.142 ng/mL (1.057–1.385), respectively (Figure 5).

## 4. Discussion

Eukaryotic cells contain mitochondria, which vary in number and shape and house multiple copies of the 16-kilobase mitochondrial genome (mtDNA). The expression and function of mtDNA are strictly coordinated with the nuclear genome. Human mtDNA is maternally inherited and encodes 13 essential components of the mitochondrial electron transport chain used for oxidative phosphorylation (OXPHOS) and ATP production [40]. Mitochondria are vital organelles in all nucleated cells, involved in various biochemical processes related to cellular respiration, metabolism, biosynthesis of macromolecules, and regulation of apoptosis, cell proliferation, and motility [41]. Consequently, it is not surprising that mitochondrial dysfunction is implicated in various human pathological conditions [42,43]. Mutations, deletions, and insertions in mtDNA play a crucial role in the pathogenesis of inherited mitochondrial diseases [38,44]. However, there are several lines of evidence supporting the idea that the alterations in mtDNA quantity can also contribute to mitochondrial and common metabolic diseases [45].

The mtDNA copy number provides a more precise measure of the number of mitochondrial genomes per cell and is closely linked to mitochondrial mass, respiratory activity, and ATP production, making it a critical indicator of overall mitochondrial health [46]. The origins of mtDNA copy number as a hallmark of dysfunction can be traced to the 1990s, when rare genetic disorders were proposed to be linked with drastic reductions in mtDNA copy number and considered as a cause of human disease. However, in several genetic mitochondrial disorders, mtDNA content is elevated. This likely reflects the upregulation of mtDNA to compensate for poor mitochondrial energetics. Successively, changes in the copy number of mtDNA were described to be related to aging [47], cancer [48], neurodegenerative diseases [49,50], diabetes [51], and other mitochondria-related diseases [52]. In this paper, we report, for the first time, an association between a decrease in mtDNA content and porphyria.

However, it is important to note that the mtDNA copy number can also change independent of disease states. In the general population, the mtDNA content measured from the peripheral blood has consistently been shown to be variable: higher in women, decreasing with age, and correlating negatively with white blood cell count [53]. Quantitative real-time PCR (qPCR) remains the most widely used and current gold standard method for measuring mtDNA copy number due to its low cost and quick turnaround time. However, recent years have seen the development of new methods using genotyping probe intensities and DNA sequencing read counts via microarray platforms and whole-exome/genome sequencing (WES/WGS), which have demonstrated accurate mtDNA content measurement. Additionally, previous studies have reported the impact of different DNA extraction methods on the accuracy of mtDNA copy number quantification, suggesting the use of more accurate organic solvent methods or direct cell lysate [54]. In this study, mtDNA was extracted from the lysate of peripheral white blood cells, and comparisons were made between two well-matched populations for sex and age to avoid confounding factors.

Our results demonstrate that a mutation in the *HMBS* gene alone was sufficient to reduce mtDNA copy number by approximately 50% compared with controls, indirectly indicating a decrease in the number of mitochondria per cell. This reduction may be a compensatory mechanism directly related to defects in the heme biosynthetic pathway in porphyria patients, leading to reduced heme production. Heme is essential for the biogenesis of the mitochondrial electron transport chain [55], and heme deficiency selectively impairs the assembly of cytochrome c oxidase (complex IV), leading to energy production deficits [56]. Dixon et al. demonstrated that AIP patients exhibit mitochondrial dysfunction, resulting in reduced mitochondrial oxygen consumption rates (OCRs), indicating limitations in electron transport and ATP production in these individuals [34]. Consistent with the hypothesis that heme deficiency contributes to a decrease in mitochondrial content, we found that AIP patients have significantly lower levels of heme compared with healthy individuals.

Heme deficiency can increase oxidative stress via impairments in the activity of complex IV, which, in turn, affects mtDNA integrity, leading to mitochondrial and neuronal decay in aging individuals [57]. The mitochondrial genome is particularly susceptible to mutations due to its high sensitivity to oxidative damage by reactive oxygen species (ROS), and mtDNA deletions are associated with a lower mtDNA copy number in various diseases [58,59]. In contrast, our cohort preserved the integrity of the mtDNA, thus suggesting that the reduction in mtDNA copy number was not directly linked to mutational damage facilitated by ROS production. While we did not assess ROS levels in this study, these results strongly suggest that constitutive heme deficiency, as seen in porphyria patients, maintains a low number of mitochondria per cell through an unknown protective regulatory mechanism designed to deactivate the non-functioning respiratory chain to prevent the consequent oxidative damage. However, the reduction in heme levels did not fully explain the differences in mtDNA levels observed between asymptomatic and symptomatic patients. Although we cannot rule out the possibility that the results were statistically insignificant due to the limited number of patients in whom heme levels were measured, we speculate that factors other than heme may contribute to this reduction.

Mitochondrial homeostasis is maintained through the coordination of two opposing processes: mitochondrial biogenesis, which generates new mitochondria, and mitophagy, which removes damaged mitochondria [60]. Given that no damaged mtDNA was detected, we hypothesized that mitochondrial biogenesis was decreased in porphyria, mainly in symptomatic patients. Mitochondrial content and respiratory capacity adapt to energy demands, and the abundance of mitochondria can be modulated by transcription coactivators of the PGC-1 family [31,61], responding to various physiological conditions and environmental stimuli, including temperature, nutritional status, and physical activity [62]. Specifically, PERM1 regulates a subset of PGC-1/ERR-target genes involved in mitochondrial bioenergetics, mediating muscle-specific mitochondrial biogenesis and oxidative capacity in response to increased energy demands [63]. Our results revealed significantly lower PERM1 levels in AIP patients, and similar to the reduction in mtDNA copy number, this impairment was more pronounced in symptomatic patients. These findings suggest that compromised mitochondrial biogenesis is the most likely cause of mtDNA reduction in AIP patients. This process, by increasing the number of mitochondria, represents a cellular response mechanism to various external stimuli and changes in physiological states that require increased rates of ATP utilization. Thus, impairment of the molecular machinery controlling mitochondrial biogenesis may influence the individual’s ability to respond to endogenous or environmental stressors, and potentially modulate the penetrance of porphyria.

Considering that the AIP phenotype only manifests in response to endogenous and exogenous triggers, these new findings suggest that patients with greater impairments in mitochondrial biogenesis may not adequately respond to environmental stimuli and physiological bioenergetic demands, experiencing symptoms. However, the metabolic and/or genetic factors responsible for this impairment need further investigation.

Furthermore, our results indicate that a higher mitochondrial DNA content is associated with decreased porphyria severity or even incomplete disease penetrance. Therefore, the mtDNA copy number in blood cells and PERM1 in serum may serve as new biomarkers to differentiate affected patients from AIP carriers, potentially enhancing the appropriate use of therapeutic approaches. In conclusion, these findings provide a new explanation for the variability in the penetrance and in clinical presentation of porphyria.

## Figures and Tables

**Table 1:** The details of participants in different groups.

	Total Number	Men	Women	Age Average	Age Median (Min-Max)
**Healthy Subjects**	37	10	27	43	44 (22–77)
**Patients**	34	16	18	40	42 (11–75)
Asymptomatic	20	10	10	39	41 (11–75)
Symptomatic	14	6	8	41	43 (19–54)

## Data Availability

Data will be available by authors on request.
